# Mental Health Outcomes in Transgender and Nonbinary Youths Receiving Gender-Affirming Care

**DOI:** 10.1001/jamanetworkopen.2022.0978

**Published:** 2022-02-25

**Authors:** Diana M. Tordoff, Jonathon W. Wanta, Arin Collin, Cesalie Stepney, David J. Inwards-Breland, Kym Ahrens

**Affiliations:** 1 Department of Epidemiology, University of Washington, Seattle; 2 Department of Psychiatry and Behavioral Sciences, University of Washington, Seattle; 3 School of Medicine, University of Washington, Seattle; 4 Department of Psychiatry and Behavioral Medicine, Department of Adolescent and Young Adult Medicine, Seattle Children’s Hospital, Seattle, Washington; 5 University of California, San Diego School of Medicine, Rady Children's Hospital; 6 Division of Adolescent Medicine, Department of Pediatrics, Seattle Children’s Hospital, Seattle, Washington

## Abstract

**Importance:**

Transgender and nonbinary (TNB) youths are disproportionately burdened by poor mental health outcomes owing to decreased social support and increased stigma and discrimination. Although gender-affirming care is associated with decreased long-term adverse mental health outcomes among these youths, less is known about its association with mental health immediately after initiation of care.

**Objective:**

To investigate changes in mental health over the first year of receiving gender-affirming care and whether initiation of puberty blockers (PBs) and gender-affirming hormones (GAHs) was associated with changes in depression, anxiety, and suicidality.

**Design, Setting, and Participants:**

This prospective observational cohort study was conducted at an urban multidisciplinary gender clinic among TNB adolescents and young adults seeking gender-affirming care from August 2017 to June 2018. Data were analyzed from August 2020 through November 2021.

**Exposures:**

Time since enrollment and receipt of PBs or GAHs.

**Main Outcomes and Measures:**

Mental health outcomes of interest were assessed via the Patient Health Questionnaire 9-item (PHQ-9) and Generalized Anxiety Disorder 7-item (GAD-7) scales, which were dichotomized into measures of moderate or severe depression and anxiety (ie, scores ≥10), respectively. Any self-report of self-harm or suicidal thoughts over the previous 2 weeks was assessed using PHQ-9 question 9. Generalized estimating equations were used to assess change from baseline in each outcome at 3, 6, and 12 months of follow-up. Bivariate and multivariable logistic models were estimated to examine temporal trends and investigate associations between receipt of PBs or GAHs and each outcome.

**Results:**

Among 104 youths aged 13 to 20 years (mean [SD] age, 15.8 [1.6] years) who participated in the study, there were 63 transmasculine individuals (60.6%), 27 transfeminine individuals (26.0%), 10 nonbinary or gender fluid individuals (9.6%), and 4 youths who responded “I don’t know” or did not respond to the gender identity question (3.8%). At baseline, 59 individuals (56.7%) had moderate to severe depression, 52 individuals (50.0%) had moderate to severe anxiety, and 45 individuals (43.3%) reported self-harm or suicidal thoughts. By the end of the study, 69 youths (66.3%) had received PBs, GAHs, or both interventions, while 35 youths had not received either intervention (33.7%). After adjustment for temporal trends and potential confounders, we observed 60% lower odds of depression (adjusted odds ratio [aOR], 0.40; 95% CI, 0.17-0.95) and 73% lower odds of suicidality (aOR, 0.27; 95% CI, 0.11-0.65) among youths who had initiated PBs or GAHs compared with youths who had not. There was no association between PBs or GAHs and anxiety (aOR, 1.01; 95% CI, 0.41, 2.51).

**Conclusions and Relevance:**

This study found that gender-affirming medical interventions were associated with lower odds of depression and suicidality over 12 months. These data add to existing evidence suggesting that gender-affirming care may be associated with improved well-being among TNB youths over a short period, which is important given mental health disparities experienced by this population, particularly the high levels of self-harm and suicide.

## Key Points

**Question:**

Is gender-affirming care for transgender and nonbinary (TNB) youths associated with changes in depression, anxiety, and suicidality?

**Findings:**

In this prospective cohort of 104 TNB youths aged 13 to 20 years, receipt of gender-affirming care, including puberty blockers and gender-affirming hormones, was associated with 60% lower odds of moderate or severe depression and 73% lower odds of suicidality over a 12-month follow-up.

**Meaning:**

This study found that access to gender-affirming care was associated with mitigation of mental health disparities among TNB youths over 1 year; given this population's high rates of adverse mental health outcomes, these data suggest that access to pharmacological interventions may be associated with improved mental health among TNB youths over a short period.

## Teaser

This cohort study investigates whether gender-affirming care is associated with decreased depression, anxiety, and suicidality among transgender and nonbinary youths.

## Introduction

Transgender and nonbinary (TNB) youths are disproportionately burdened by poor mental health outcomes, including depression, anxiety, and suicidal ideation and attempts.^1,2,3,4,5^ These disparities are likely owing to high levels of social rejection, such as a lack of support from parents^6,7^ and bullying,^6,8,9^ and increased stigma and discrimination experienced by TNB youths. Multidisciplinary care centers have emerged across the country to address the health care needs of TNB youths, which include access to medical gender-affirming interventions, such as puberty blockers (PBs) and gender-affirming hormones (GAHs).^10^ These centers coordinate care and help youths and their families address barriers to care, such as lack of insurance coverage^11^ and travel times.^12^ Gender-affirming care is associated with decreased rates of long-term adverse outcomes among TNB youths. Specifically, PBs, GAHs, and gender-affirming surgeries have all been found to be independently associated with decreased rates of depression, anxiety, and other adverse mental health outcomes.^13,14,15,16^ Access to these interventions is also associated with a decreased lifetime incidence of suicidal ideation among adults who had access to PBs during adolescence.^17^ Conversely, TNB youths who present to care later in adolescence or young adulthood experience more adverse mental health outcomes.^18^ Despite this robust evidence base, legislation criminalizing and thus limiting access to gender-affirming medical care for minors is increasing.^19,20^

Less is known about the association of gender-affirming care with mental health outcomes immediately after initiation of care. Several studies published from 2015 to 2020 found that receipt of PBs or GAHs was associated with improved psychological functioning^21^ and body satisfaction,^22^ as well as decreased depression^23^ and suicidality^24^ within a 1-year period. Initiation of gender-affirming care may be associated with improved short-term mental health owing to validation of gender identity and clinical staff support. Conversely, prerequisite mental health evaluations, often perceived as pathologizing by TNB youths, and initiation of GAHs may present new stressors that may be associated with exacerbation of mental health symptoms early in care, such as experiences of discrimination associated with more frequent points of engagement in a largely cisnormative health care system (eg, interactions with nonaffirming pharmacists to obtain laboratory tests, syringes, and medications).^25^ Given the high risk of suicidality among TNB adolescents, there is a pressing need to better characterize mental health trends for TNB youths early in gender-affirming care. This study aimed to investigate changes in mental health among TNB youths enrolled in an urban multidisciplinary gender clinic over the first 12 months of receiving care. We also sought to investigate whether initiation of PBs or GAHs was associated with depression, anxiety, and suicidality.

## Methods

This cohort study received approval from the Seattle Children’s Hospital Institutional Review Board. For youths younger than age 18 years, caregiver consent and youth assent was obtained. For youths ages 18 years and older, youth consent alone was obtained. The 12-month assessment was funded via a different mechanism than other survey time points; thus, participants were reconsented for the 12-month survey. The study follows the Strengthening the Reporting of Observational Studies in Epidemiology (STROBE) reporting guideline.

### Study Procedures

We conducted a prospective observational cohort study of TNB youths seeking care at Seattle Children’s Gender Clinic, an urban multidisciplinary gender clinic. After a referral is placed or a patient self-refers, new patients, their caregivers, or patients with their caregivers are scheduled for a 1-hour phone intake with a care navigator who is a licensed clinical social worker. Patients are then scheduled for an appointment at the clinic with a medical provider.

All patients who completed the phone intake and in-person appointment between August 2017 and June 2018 were recruited for this study. Participants completed baseline surveys within 24 hours of their first appointment and were invited to complete follow-up surveys at 3, 6, and 12 months. Youth surveys were used to assess most variables in this study; caregiver surveys were used to assess caregiver income. Participation and completion of study surveys had no bearing on prescribing of PBs or GAHs.

### Measures

#### Mental Health Variables

We assessed 3 internalizing mental health outcomes: depression, generalized anxiety, and suicidality. Depression was assessed using the Patient Health Questionnaire 9-item scale (PHQ-9), and anxiety was assessed using the Generalized Anxiety Disorder 7-item scale (GAD-7). We dichotomized PHQ-9 and GAD-7 scores into measures of moderate or severe depression and anxiety (ie, scores ≥10).^26,27^ Self-harm and suicidal thoughts were assessed using PHQ-9 question 9 (eTable 1 in the Supplement).

#### Pharmacological Interventions

Participants self-reported if they had ever received GAHs, including estrogen or testosterone, or PBs (eg, gonadotropin-releasing hormone analogues) on each survey. We conducted a medical record review to capture prescription of androgen blockers (eg, spironolactone) and medications for menstrual suppression or contraception (ie, medroxyprogesterone acetate or levonorgestrel-releasing intrauterine device) during the study period.

#### Covariates

We a priori considered potential confounders hypothesized to be associated with our exposures and outcomes of interest based on theory and prior research. Self-reported gender was ascertained on each survey using a 2-step question that asked participants about their current gender and their sex assigned at birth. If a participant’s self-reported gender changed across surveys, we used the gender reported most frequently by a participant (3 individuals identified as transmasculine at baseline and as nonbinary on all follow-up surveys). We collected data on self-reported race and ethnicity (available response options were Arab or Middle Eastern; Asian; Black or African American; Latinx; Native American, American Indian, or Alaskan Native or Native Hawaiian; Pacific Islander; and White), age, caregiver income, and insurance type. Race and ethnicity were assessed as potential covariates owing to known barriers to accessing gender-affirming care among transgender youth who are members of minority racial and ethnic groups. For descriptive statistics, Asian and Pacific Islander groups were combined owing to small population numbers. We included a baseline variable reflecting receipt of ongoing mental health therapy other than for the purpose of a mental health assessment to receive a gender dysphoria diagnosis. We included a self-report variable reflecting whether youths felt their gender identity or expression was a source of tension with their parents or guardians. Substance use included any alcohol, marijuana, or other drug use in the past year. Resilience was measured by the Connor-Davidson Resilience Scale (CD-RISC) 10-item score developed to measure change in an individual’s state resilience over time.^28^ Resilience scores were dichotomized into high (ie, ≥median) and low (ie, <median). Prior studies of young adults in the US reported mean CD-RISC scores ranging from 27.2 to 30.1.^29,30^

### Statistical Analysis

We used generalized estimating equations to assess change in outcomes from baseline at each follow-up point (eFigure 1 in the Supplement). We used a logit link function to estimate adjusted odds ratio (aOR) for the association between variables and each mental health outcome. We initially estimated bivariate associations between potential confounders and mental health outcomes. Multivariable models included variables that were statistically significant in bivariate models. For all outcomes and models, statistical significance was defined as 95% CIs that did not contain 1.00. Reported *P* values are based on 2-sided Wald test statistics.

Model 1 examined temporal trends in mental health outcomes, with time (ie, baseline, 3, 6, and 12 months) modeled as a categorical variable. Model 2 estimated the association between receipt of PBs or GAHs and mental health outcomes adjusted for temporal trends and potential confounders. Receipt of PBs or GAHs was modeled as a composite binary time-varying exposure that compared mean outcomes between participants who had initiated PBs or GAHs and those who had not across all time points (eTable 2 in the Supplement). All models used an independent working correlation structure and robust standard errors to account for the time-varying exposure variable.

We performed several sensitivity analyses. Because our data were from an observational cohort, we first considered the degree to which they were sensitive to unmeasured confounding. To do this, we calculated the E-value for the association between PBs or GAHs and mental health outcomes in model 2. The E-value is defined as the minimum strength of association that a confounder would need to have with both exposure and outcome to completely explain away their association (eTable 4 in the Supplement).^31^ Second, we performed sensitivity analyses on several subsets of youths. We separately examined the association of PBs and GAHs with outcomes of interest, although we a priori did not anticipate being powered to detect statistically significant outcomes owing to our small sample size and the relatively low proportion of youths who accessed PBs. We also conducted sensitivity analyses using the Patient Health Questionnaire 8-item scale (PHQ-8), in which the PHQ-9 question 9 regarding self-harm or suicidal thoughts was removed, given that we analyzed this item as a separate outcome. Lastly, we restricted our analysis to minor youths ages 13 to 17 years because they were subject to different laws and policies related to consent and prerequisite mental health assessments. We used R statistical software version 3.6.2 (R Project for Statistical Computing) to conduct all analyses. Data were analyzed from August 2020 through November 2021.

## Results

A total of 169 youths were screened for eligibility during the study period, among whom 161 eligible youths were approached. Nine youths or caregivers declined participation, and 39 youths did not complete consent or assent or did not complete the baseline survey, leaving a sample of 113 youths (70.2% of approached youths). We excluded 9 youths aged younger than 13 years from the analysis because they received different depression and anxiety screeners. Our final sample included 104 youths ages 13 to 20 years (mean [SD] age, 15.8 [1.6] years). Of these individuals, 84 youths (80.8%), 84 youths, and 65 youths (62.5%) completed surveys at 3, 6, and 12 months, respectively.

Our cohort included 63 transmasculine youths (60.6%), 27 transfeminine youths (26.0%), 10 nonbinary or gender fluid youths (9.6%), and 4 youths who responded “I don’t know” or did not respond to the gender identity question on all completed questionnaires (3.8%) (Table 1). There were 4 Asian or Pacific Islander youths (3.8%), 3 Black or African American youths (2.9%); 9 Latinx youths (8.7%); 6 Native American, American Indian, or Alaskan Native or Native Hawaiian youths (5.8%); 67 White youths (64.4%); and 9 youths who reported more than 1 race or ethnicity (8.7%). Race and ethnicity data were missing for 6 youth (5.8%).

**Table 1.** Participant Characteristics

Characteristic	Participants, No. (%) (N = 104)
Gender	
Male or transgender male	63 (60.6)
Female or transgender female	27 (26.0)
Nonbinary or gender fluid	10 (9.6)
Don't know or missing	4 (3.8)
Race and ethnicity^a^	
Asian or Pacific Islander	4 (3.8)
Black or African American	3 (2.9)
Latinx	9 (8.7)
Native American, American Indian, or Alaskan Native or Native Hawaiian	6 (5.8)
White	67 (64.4)
More than 1 race or ethnicity chosen	9 (8.7)
Missing	6 (5.8)
Age at baseline, y	
13	8 (7.7)
14	20 (19.2)
15	18 (17.3)
16	22 (21.2)
17	22 (21.2)
18	8 (7.7)
19	5 (4.8)
20	1 (1.0)
Pharmacological intervention	
PBs^b^	19 (18.2)
GAHs^b^	64 (61.5)
Androgen blockers^c^	17 (51.5)
Menstrual suppression or contraception^d^	25 (35.2)
Depression at baseline (using PHQ-9)	
0-4 (minimal)	14 (13.5)
5-9 (mild)	27 (26.0)
10-14 (moderate)	22 (21.2)
15-19 (moderately severe)	11 (10.6)
≥20 (severe)	26 (25.0)
Missing	4 (3.8)
Anxiety at baseline (using GAD-7)	
0-4 (minimal)	20 (19.2)
5-9 (mild)	28 (26.9)
10-14 (moderate)	20 (19.2)
≥15 (severe)	32 (30.8)
Missing	4 (3.8)
Self-harm or suicidal thoughts at baseline	45 (43.2)
Receiving mental health therapy	65 (62.5)
Tension with caregiver about gender identity or expression	36 (34.6)
Any substance use	34 (32.7)
Resilience at baseline (using CD-RISC 10)	
0-10	8 (7.7)
10-20	35 (33.7)
21-30	15 (14.4)
30-40	34 (32.7)
Missing	12 (11.5)

Abbreviations: CD-RISC 10, Connor-Davidson 10-item Resilience Scale; GAD-7, Generalized Anxiety Disorder 7-item scale; GAH, gender-affirming hormone; PB, puberty blocker; PHQ-9 Patient Health Questionnaire 9-item scale.

^a^ Available response options for race and ethnicity were Arab or Middle Eastern; Asian or Pacific Islander; Black or African American; Latinx; Native American, American Indian, or Alaskan Native or Native Hawaiian; Pacific Islander; and White. Asian and Pacific Islander groups were combined owing to small population sizes.

^b^ Self-reported receipt ever of PBs or GAHs at baseline or through the end of the study period.

^c^ Includes androgen blockers received during the study period; percentage is among 33 youths assigned male sex at birth.

^d^ Includes pharmacological interventions for menstrual suppression or contraception received during the study period; percentage is among 71 youths assigned female sex at birth.

At baseline, 7 youths had ever received PBs or GAHs (including 1 youth who received PBs, 4 youths who received GAHs, and 2 youths who received both PBs and GAHs). By the end of the study, 69 youths (66.3%) had received PBs or GAHs (including 50 youths who received GAHs only [48.1%], 5 youths who received PBs only [4.8%], and 14 youths who received PBs and GAHs [13.5%]), while 35 youths had not received either PBs or GAHs (33.7%) (eTable 3 in the Supplement). Among 33 participants assigned male sex at birth, 17 individuals (51.5%) had received androgen blockers, and among 71 participants assigned female sex at birth, 25 individuals (35.2%) had received menstrual suppression or contraceptives by the end of the study.

A large proportion of youths reported depressive and anxious symptoms at baseline. Specifically, 59 individuals (56.7%) had baseline PHQ-9 scores of 10 or more, suggesting moderate to severe depression; there were 22 participants (21.2%) scoring in the moderate range, 11 participants (10.6%) in the moderately severe range, and 26 participants (25.0%) in the severe range. Similarly, half of participants had a GAD-7 score suggestive of moderate to severe anxiety at baseline (52 individuals [50.0%]), including 20 participants (19.2%) scored in the moderate range, and 32 participants (30.8%) scored in the severe range. There were 45 youths (43.3%) who reported self-harm or suicidal thoughts in the prior 2 weeks. At baseline, 65 youths (62.5%) were receiving ongoing mental health therapy, 36 youths (34.6%) reported tension with their caregivers about their gender identity or expression, and 34 youths (32.7%) reported any substance use in the prior year. Lastly, we observed a wide range of resilience scores (median [range], 22.5 [1-38], with higher scores equaling more resiliency). There were no statistically significant differences in baseline characteristics by gender.

In bivariate models, substance use was associated with all mental health outcomes (Table 2). Youths who reported any substance use were 4-fold as likely to have PHQ-9 scores of moderate to severe depression (aOR, 4.38; 95% CI, 2.10-9.16) and 2-fold as likely to have GAD-7 scores of moderate to severe anxiety (aOR, 2.07; 95% CI, 1.04-4.11) or report thoughts of self-harm or suicide in the prior 2 weeks (aOR, 2.06; 95% CI, 1.08-3.93). High resilience scores (ie, ≥median), compared with low resilience scores (ie, <median), were associated with lower odds of moderate or severe anxiety (aOR, 0.51; 95% CI, 0.26-0.999).

**Table 2.** Baseline Factors Associated With Mental Health Outcomes in Bivariate Models

Factor	Moderate or severe depression (PHQ-9 ≥10)^a^		Moderate or severe anxiety (GAD-7 ≥10)^b^		Any self-harm or suicidal thoughts^c^	
	aOR (95% CI)	*P* value	aOR (95% CI)	*P* value	aOR (95% CI)	*P* value
PBs or GAHs	0.67 (0.33-1.34)	.25	0.90 (0.49-1.66)	.74	0.47 (0.26-0.86)	.01
Time, mo						
0 (baseline)	1 [Reference]	NA	1 [Reference]	NA	1 [Reference]	NA
3	1.96 (0.99-3.90)	.05	1.46 (0.71-2.97)	.30	1.00 (0.49-2.06)	.99
6	1.01 (0.46-2.19)	.99	0.77 (0.39-1.52)	.45	1.22 (0.64-2.34)	.54
12	1.42 (0.55-3.66)	.47	0.95 (0.43-2.06)	.89	1.02 (0.41-2.52)	.97
Gender						
Male or transgender male	1 [Reference]	NA	1 [Reference]	NA	1 [Reference]	NA
Female or transgender female	1.07 (0.51-2.24)	.87	3.15 (0.92-10.8)	.07	1.20 (0.55-2.64)	.64
Nonbinary or gender fluid	2.40 (0.84-6.87)	.10	1.35 (0.67-2.72)	.40	2.17 (0.73-6.41)	.16
Race or ethnicity						
White	1 [Reference]	NA	1 [Reference]	NA	1 [Reference]	NA
Member of minority race or ethnic group^d^	1.08 (0.51-2.28)	.84	0.86 (0.45-1.66)	.66	0.92 (0.53-1.61)	.77
Age, y						
13-15	1 [Reference]	NA	1 [Reference]	NA	1 [Reference]	NA
16-17	1.79 (0.82-3.88)	.14	0.63 (0.29-1.39)	.25	0.86 (0.44-1.68)	.66
18-20	0.78 (0.24-2.51)	.68	1.17 (0.43-3.17)	.76	0.79 (0.36-1.74)	.55
Mental health and substance use at baseline						
Moderate or severe depression (PHQ-9 ≥10)	27.2 (13.4-55.4)	<.001	1.91 (0.85-4.29)	.12	1.06 (0.50-2.24)	.88
Moderate or severe anxiety (GAD-7 ≥10)	4.90 (2.27-10.6)	<.001	14.3 (7.31-27.9)	<.001	1.44 (0.76-2.72)	.27
Self-harm or suicidal thoughts	1.32 (0.61-2.85)	.48	1.49 (0.73-3.06)	.28	18.9 (10.4-34.1)	<.001
Receiving mental health therapy	1.46 (0.69-3.08)	.32	0.65 (0.31-1.38)	.26	0.75 (0.36-1.56)	.45
Tension with caregivers about gender identity or expression	1.93 (0.90-4.14)	.09	1.06 (0.52-2.15)	.87	1.55 (0.88-2.74)	.13
Any substance use	4.38 (2.10-9.16)	<.001	2.07 (1.04-4.11)	.04	2.06 (1.08-3.93)	.03
Resilience at baseline (CD-RISC 10 ≥22.5)^e^	0.85 (0.42-1.74)	.67	0.51 (0.26-1.00)	.05	0.74 (0.39-1.44)	.38

Abbreviations: aOR, adjusted odds ratio; CD-RISC 10, Connor-Davidson 10-item Resilience Scale; GAD-7, Generalized Anxiety Disorder 7-item scale; GAH, gender-affirming hormone; NA, not applicable; PB, puberty blocker; PHQ-9, Patient Health Questionnaire 9-item scale.

^a^ Bivariate models are adjusted for baseline PHQ-9.

^b^ Bivariate models are adjusted for baseline GAD-7.

^c^ Bivariate models are adjusted for self-harm or suicidal thoughts reported at baseline.

^d^ Owing to small sample sizes, this group includes Asian or Pacific Islander; Black or African American; Latinx; and Native American, American Indian, Alaskan Native, or Native Hawaiian youths and youths who reported more than 1 race or ethnicity.

^e^ The median (range) CD-RISC score for the cohort was 22.5 (1-38).

There were no statistically significant temporal trends in the bivariate model or model 1 (Table 2 and Table 3). However, among all participants, odds of moderate to severe depression increased at 3 months of follow-up relative to baseline (aOR, 2.12; 95% CI, 0.98-4.60), which was not a significant increase, and returned to baseline levels at months 6 and 12 (Figure) prior to adjusting for receipt of PBs or GAHs.

**Table 3.** Temporal Trends in Mental Health Outcomes in Multivariable Model 1^a^

Factor	Moderate or severe depression (PHQ-9 ≥10)		Moderate or severe anxiety (GAD-7 ≥10)		Any self-harm or suicidal thoughts	
	aOR (95% CI)	*P* value	aOR (95% CI)	*P* value	aOR (95% CI)	*P* value
Time, mo						
0 (baseline)	1 [Reference]	NA	1 [Reference]	NA	1 [Reference]	NA
3	2.12 (0.98-4.60)	.06	1.50 (0.71-3.15)	.29	0.99 (0.48-2.06)	.98
6	0.99 (0.42-2.35)	.98	0.78 (0.38-1.59)	.49	1.22 (0.63-2.36)	.56
12	1.27 (0.44-3.67)	.66	0.96 (0.43-2.11)	.91	0.98 (0.39-2.48)	.97
Mental health and substance use at baseline						
Moderate or severe depression (PHQ-9 ≥10)	18.5 (8.44-40.5)	<.001	NA	NA	NA	NA
Moderate or severe anxiety (GAD-7 ≥10)	3.63 (1.83-7.19)	<.001	12.4 (6.25-24.7)	<.001	NA	NA
Self-harm or suicidal thoughts	NA	NA	NA	NA	19.9 (10.9-36.1)	<.001
Any substance use	3.35 (1.56-7.18)	.002	2.21 (1.09-4.49)	.03	2.07 (1.09-3.93)	.03
Resilience at Baseline (CD-RISC 10 ≥22.5)^b^	NA	NA	0.48 (0.24-0.95)	.04	NA	NA

Abbreviations: aOR, adjusted odds ratio; CD-RISC 10, Connor-Davidson 10-item Resilience Scale; GAD-7, Generalized Anxiety Disorder 7-item scale; NA, not applicable; PHQ-9, Patient Health Questionnaire 9-item scale.

^a^ Model 1 includes categorical temporal variables (ie, months 3, 6, and 12 relative to baseline) and covariates that were statistically significant in bivariate models (such that 95% CIs did not contain 1.00) (see Table 2). Covariates that were not significant in bivariate models are marked NA.

^b^ The median (range) CD-RISC score for the cohort is 22.5 (1-38).

**Figure.**
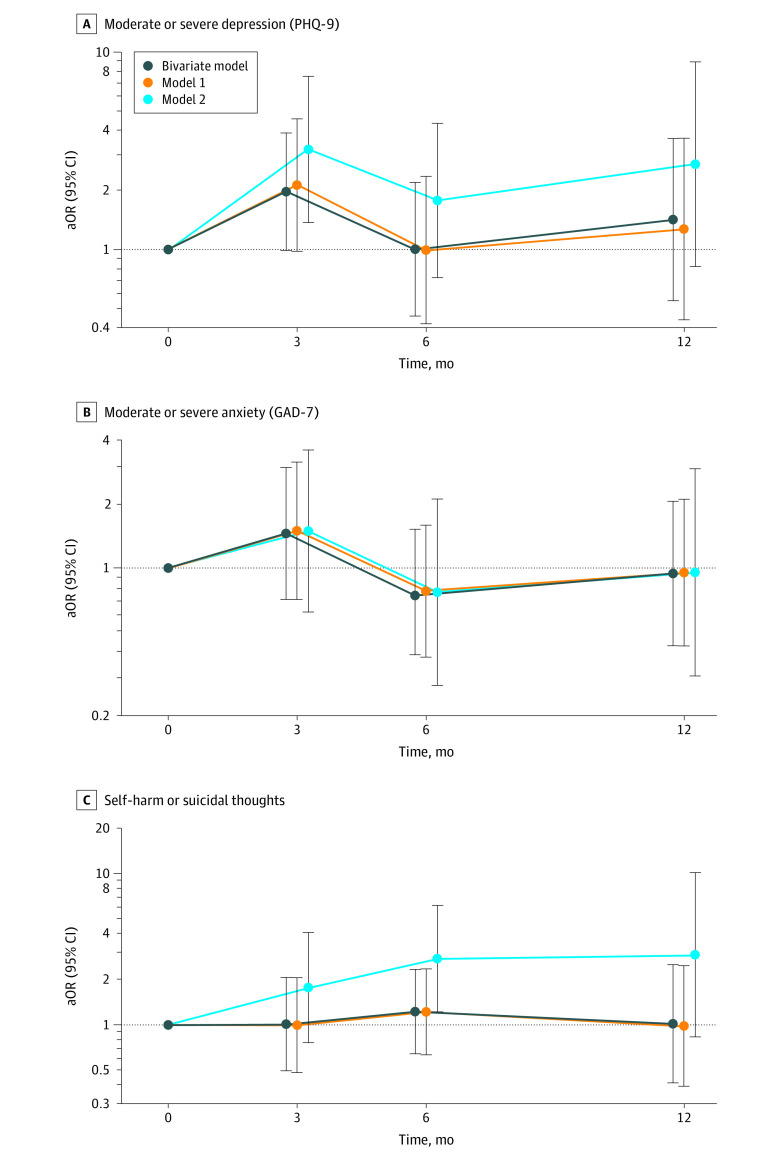
Temporal Trends in Mental Health Outcomes Outcomes are estimated from bivariate and multivariable generalized estimating equation models. aOR, indicates adjusted odds ratio; GAD-7, Generalized Anxiety Disorder 7-item scale; PHQ-9, Patient Health Questionnaire 9-item scale; whiskers, 95% CIs.

We also examined the association between receipt of PBs or GAHs and mental health outcomes in bivariate and multivariable models (eFigure 2 in the Supplement). After adjusting for temporal trends and potential confounders (Table 4), we observed that youths who had initiated PBs or GAHs had 60% lower odds of moderate to severe depression (aOR, 0.40; 95% CI, 0.17-0.95) and 73% lower odds of self-harm or suicidal thoughts (aOR, 0.27; 95% CI, 0.11-0.65) compared with youths who had not yet initiated PBs or GAHs. There was no association between receipt of PBs or GAHs and moderate to severe anxiety (aOR, 1.01; 95% CI, 0.41-2.51). After adjusting for time-varying exposure of PBs or GAHs in model 2 (Table 4), we observed statistically significant increases in moderate to severe depression among youths who had not received PBs or GAHs by 3 months of follow-up (aOR, 3.22; 95% CI, 1.37-7.56). A similar trend was observed for self-harm or suicidal thoughts among youths who had not received PBs or GAHs by 6 months of follow-up (aOR, 2.76; 95% CI, 1.22-6.26). Lastly, we estimated E-values of 2.56 and 3.25 for the association between receiving PGs or GAHs and moderate to severe depression and suicidality, respectively (eTable 4 in the Supplement). Sensitivity analyses obtained comparable results and are presented in eTables 5 through 8 in the Supplement.

**Table 4.** Association Between GAHs or PBs and Mental Health Outcomes in Multivariable Model 2^a^

Factor	Moderate or severe depression (PHQ-9 ≥10)		Moderate or severe anxiety (GAD-7≥10)		Any self-harm or suicidal thoughts	
	aOR (95% CI)	*P* value	aOR (95% CI)	*P* value	aOR (95% CI)	*P* value
PBs or GAHs	0.40 (0.17-0.95)	.04	1.01 (0.41-2.51)	.98	0.27 (0.11-0.65)	.003
Time, mo						
0 (baseline)	1 [Reference]	NA	1 [Reference]	NA	1 [Reference]	NA
3 mo	3.22 (1.37-7.56)	.007	1.49 (0.62-3.59)	.37	1.77 (0.76-4.13)	.19
6 mo	1.77 (0.72-4.37)	.21	0.77 (0.28-2.11)	.61	2.76 (1.22-6.26)	.02
12 mo	2.71 (0.82-8.95)	.10	0.95 (0.31-2.93)	.93	2.93 (0.83-10.4)	.10
Mental health & substance use at baseline						
Moderate or severe depression (PHQ-9 ≥10)	19.4 (8.64-43.4)	<.001	NA	NA	NA	NA
Moderate or severe anxiety (GAD-7 ≥10)	3.82 (1.87-7.82)	<.001	12.4 (6.25-24.7)	<.001	NA	NA
Self-harm or suicidal thoughts	NA	NA	NA	NA	23.9 (12.9-44.5)	<.001
Any substance use	3.20 (1.49-6.84)	.003	2.21 (1.09-4.50)	.03	2.00 (1.08-3.73)	.03
Resilience at baseline (CD-RISC 10 ≥22.5)^b^	NA	NA	0.48 (0.24-0.95)	.04	NA	NA

Abbreviations: aOR, adjusted odds ratio; CD-RISC 10, Connor-Davidson 10-item Resilience Scale; GAD-7, Generalized Anxiety Disorder 7-item scale; GAH, gender-affirming hormone; NA, not applicable; PB, puberty blocker; PHQ-9, Patient Health Questionnaire 9-item scale.

^a^ Model 2 includes a time-varying exposure variable measuring the receipt of PBs or GAHs adjusted for temporal trend (ie, categorical variable for months 3, 6, and 12 relative to baseline) and covariates that were statistically significant in the bivariate models (such that 95% CIs did not contain 1.00) (see Table 2). The unadjusted bivariate associations between PBs or GAHs and mental health outcomes are reported in Table 2. Covariates that were not significant in bivariate models are marked NA.

^b^ The median (range) CD-RISC score for the cohort is 22.5 (1-38).

## Discussion

In this prospective clinical cohort study of TNB youths, we observed high rates of moderate to severe depression and anxiety, as well as suicidal thoughts. Receipt of gender-affirming interventions, specifically PBs or GAHs, was associated with 60% lower odds of moderate to severe depressive symptoms and 73% lower odds of self-harm or suicidal thoughts during the first year of multidisciplinary gender care. Among youths who did not initiate PBs or GAHs, we observed that depressive symptoms and suicidality were 2-fold to 3-fold higher than baseline levels at 3 and 6 months of follow-up, respectively. Our study results suggest that risks of depression and suicidality may be mitigated with receipt of gender-affirming medications in the context of a multidisciplinary care clinic over the relatively short time frame of 1 year.

Our findings are consistent with those of prior studies finding that TNB adolescents are at increased risk of depression, anxiety, and suicidality^1,11,32^ and studies finding long-term and short-term improvements in mental health outcomes among TNB individuals who receive gender-affirming medical interventions.^14,21,22,23,24,33,34^ Surprisingly, we observed no association with anxiety scores. A recent cohort study of TNB youths in Dallas, Texas, found that total anxiety symptoms improved over a longer follow-up of 11 to 18 months; however, similar to our study, the authors did not observe statistically significant improvements in generalized anxiety.^22^ This suggests that anxiety symptoms may take longer to improve after the initiation of gender-affirming care. In addition, Olson et al^35^ found that prepubertal TNB children who socially transitioned did not have increased rates of depression symptoms but did have increased rates of anxiety symptoms compared with children who were cisgender. Although social transition and access to gender-affirming medical care do not always go hand in hand, it is noteworthy that access to gender-affirming medical care and supported social transition appear to be associated with decreased depression and suicidality more than anxiety symptoms.

Time trends were not significant in our study; however, it is important to note that we observed a transient and nonsignificant worsening in mental health outcomes in the first several months of care among all participants and that these outcomes subsequently returned to baseline by 12 months. This is consistent with findings from a 2020 study^36^ in an academic medical center in the northwestern US that observed no change in TNB adolescents’ GAD-7 or PHQ-9 scores from intake to first follow-up appointment, which occurred a mean of 4.7 months apart. Given that receipt of PBs or GAHs was associated with protection against depression and suicidality in our study, it could be that delays in receipt of medications is associated with initially exacerbated mental health symptoms that subsequently improve. It is also possible that mental health improvements associated with receiving these interventions may have a delayed onset, given the delay in physical changes after starting GAHs.

Few of our hypothesized confounders were associated with mental health outcomes in this sample, most notably receipt of ongoing mental health therapy and caregiver support; however, this is not surprising given that these variables were colinear with baseline mental health, which we adjusted for in all models. Substance use was the only variable associated with all mental health outcomes. In addition, youths with high baseline resilience scores were half as likely to experience moderate to severe anxiety as those with low scores. This finding suggests that substance use and resilience may be additional modifiable factors that could be addressed through multidisciplinary gender-affirming care. We recommend more granular assessment of substance use and resilience to better understand support needs (for substance use) and effective support strategies (for resilience) for TNB youths in future research.

This study has a number of strengths. This is one of the first studies to quantify a short-term transient increase in depressive symptoms experienced by TNB youths after initiating gender-affirming care, a phenomenon observed clinically by some of the authors and described in qualitative research.^37^ Although we are unable to make causal statements owing to the observational design of the study, the strength of associations between gender-affirming medications and depression and suicidality, with large aOR values, and sensitivity analyses that suggest that these findings are robust to moderate levels of unmeasured confounding. Specifically, E-values calculated for this study suggest that the observed associations could be explained away only by an unmeasured confounder that was associated with both PBs and GAHs and the outcomes of interest by a risk ratio of 2-fold to 3-fold each, above and beyond the measured confounders, but that weaker confounding could not do so.^31^

### Limitations

Our findings should be interpreted in light of the following limitations. This was a clinical sample of TNB youths, and there was likely selection bias toward youths with supportive caregivers who had resources to access a gender-affirming care clinic. Family support and access to care are associated with protection against poor mental health outcomes, and thus actual rates of depression, anxiety, and suicidality in nonclinical samples of TNB youths may differ. Youths who are unable to access gender-affirming care owing to a lack of family support or resources require particular emphasis in future research and advocacy. Our sample also primarily included White and transmasculine youths, limiting the generalizability of our findings. In addition, the need to reapproach participants for consent and assent for the 12-month survey likely contributed to attrition at this time point. There may also be residual confounding because we were unable to include a variable reflecting receipt of psychotropic medications that could be associated with depression, anxiety, and self-harm and suicidal thought outcomes. Additionally, we used symptom-based measures of depression, anxiety, and suicidality; further studies should include diagnostic evaluations by mental health practitioners to track depression, anxiety, gender dysphoria, suicidal ideation, and suicide attempts during gender care.^2^

## Conclusions

Our study provides quantitative evidence that access to PBs or GAHs in a multidisciplinary gender-affirming setting was associated with mental health improvements among TNB youths over a relatively short time frame of 1 year. The associations with the highest aORs were with decreased suicidality, which is important given the mental health disparities experienced by this population, particularly the high levels of self-harm and suicide. Our findings have important policy implications, suggesting that the recent wave of legislation restricting access to gender-affirming care^19^ may have significant negative outcomes in the well-being of TNB youths.^20^ Beyond the need to address antitransgender legislation, there is an additional need for medical systems and insurance providers to decrease barriers and expand access to gender-affirming care.

## Notes

Supplement. **eTable 1.** Survey Instruments **eTable 2.** Prevalence of Exposure Over Time **eTable 3.** Prevalence of Outcomes Over Time by Exposure Group **eTable 4.** E-Value Calculation for Association Between Puberty Blockers or Gender-Affirming Hormones and Mental Health Outcomes **eTable 5.** Examining Association Between Puberty Blockers or Gender-Affirming Hormones and Mental Health Outcomes Separately **eTable 6.** Bivariate Model Restricted to Youths Ages 13 to 17 Years **eTable 7.** Multivariable Model Restricted to 90 Youths Ages 13 to 17 Years **eTable 8.** Sensitivity Analyses using Patient Health Questionnaire 8-item Scale Score of 10 or Greater for Moderate to Severe Depression **eFigure 1.** Schematic of Generalized Estimating Equation Model **eFigure 2.** Association Between Receipt of Gender-Affirming Hormones or Puberty Blockers and Mental Health Outcomes
eReferences
