# Data Errors in eTables 2 and 3

**DOI:** 10.1001/jamanetworkopen.2022.29031

**Published:** 2022-07-26

The Original Investigation, “Mental Health Outcomes in Transgender and Nonbinary Youths Receiving Gender-Affirming Care,”^1^ published on February 25, 2022, in *JAMA Network Open*, had minor errors in the numbers of patients in eTables 2 and 3 in the Supplement; several of the numbers were missing 1 patient. These numbers and corresponding percentages have been corrected.^1^

## References

[1] Tordoff DM, Wanta JW, Collin A, Stepney C, Inwards-Breland DJ, Ahrens K. Mental health outcomes in transgender and nonbinary youths receiving gender-affirming care. JAMA Netw Open. 2022;5(2):e220978. doi:10.1001/jamanetworkopen.2022.0978 35212746 PMC8881768

