# Supplementary material for: Mental Health Outcomes in Transgender and Nonbinary Youths Receiving Gender-Affirming Care
Source: JAMA Netw Open. 2022 Feb 25;5(2):e220978. doi: 10.1001/jamanetworkopen.2022.0978 (PMC8881768; doi:10.1001/jamanetworkopen.2022.0978)
Supplement: Supplement. — eTable 1. Survey Instruments eTable 2. Prevalence of Exposure Over Time eTable 3. Prevalence of Outcomes Over Time by Exposure Group eTable 4. E-Value Calculation for Association Between Puberty Blockers or Gender-Affirming Hormones and Mental Health Outcomes eTable 5. Examining Association Between Puberty Blockers or Gender-Affirming Hormones and Mental Health Outcomes Separately eTable 6. Bivariate Model Restricted to Youths Ages 13 to 17 Years eTable 7. Multivariable Model Restricted to 90 Youths Ages 13 to 17 Years eTable 8. Sensitivity Analyses using Patient Health Questionnaire 8-item Scale Score of 10 or Greater for Moderate to Severe Depression eFigure 1. Schematic of Generalized Estimating Equation Model eFigure 2. Association Between Receipt of Gender-Affirming Hormones or Puberty Blockers and Mental Health Outcomes eReferences [file jamanetwopen-e220978-s001.pdf]

Supplemental Online Content

Tordoff DM, Wanta JW, Collin A, Stepney C, Inwards-Breland DJ, Ahrens K. Mental health outcomes in transgender and nonbinary youths receiving gender-affirming care. *JAMA Netw Open*. 2022;5(2):e220978. doi:10.1001/jamanetworkopen.2022.0978

eTable 1. Survey Instruments
eTable 2. Prevalence of Exposure Over Time
eTable 3. Prevalence of Outcomes Over Time by Exposure Group
eTable 4. E-Value Calculation for Association Between Puberty Blockers or Gender-Affirming Hormones and Mental Health Outcomes
eTable 5. Examining Association Between Puberty Blockers or Gender-Affirming Hormones and Mental Health Outcomes Separately
eTable 6. Bivariate Model Restricted to Youths Ages 13 to 17 Years
eTable 7. Multivariable Model Restricted to 90 Youths Ages 13 to 17 Years
eTable 8. Sensitivity Analyses Using Patient Health Questionnaire 8-item Scale Score of 10 or Greater for Moderate to Severe Depression
eFigure 1. Schematic of Generalized Estimating Equation Model
eFigure 2. Association Between Receipt of Gender-Affirming Hormones or Puberty Blockers and Mental Health Outcomes
eReferences

This supplemental material has been provided by the authors to give readers additional information about their work.

© 2022 Tordoff DM et al. *JAMA Network Open.*

I. Measures and Survey Instruments

Below we include the exact survey instruments used to ascertain gender, exposure variables, mental health outcome variables, and covariates from youth on the baseline, 3, 6, and 12 month follow-up surveys.

**eTable 1.** Survey Instruments	
Demographics	
Two-step Gender Identity Question	1. What is your gender identity?<br>o Transgender male (female to male)<br>o Transgender female (male to female)<br>o Male<br>o Female<br>o Non-binary or gender fluid<br>o Other: [open text box]<br>o I don't know<br>2. What sex were you assigned at birth?<br>o Male<br>o Female
Exposure Measures	
Puberty Blockers	Puberty blockers are a medication that put a young person's puberty development on pause. Have you taken puberty blockers?<br>• Yes<br>• No<br>• I don't know
Gender-affirming Hormones	Have you taken cross-sex hormones (testosterone or estrogen)?<br>• Yes<br>• No<br>• I don't know
Mental Health Outcome Measures	
Generalized Anxiety Disorder 7-item scale (GAD-7)	Over the last 2 weeks, how often have you been bothered by the following problems?<br>1. Feeling nervous, anxious, or on edge<br>2. Not being able to stop or control worrying<br>3. Worrying to much about different things<br>4. Trouble relaxing<br>5. Being so restless that it's hard to sit still<br>6. Becoming easily annoyed or irritable<br>7. Feeling afraid as if something awful might happen<br>With response options: not at all, several days, over half of days, nearly everyone day, and I don't know.<br><br>8. If you checked off any problems, how difficult have these made it for you to do your work, take care of things at home, or get along with people?<br>o Not difficult at all<br>o Somewhat difficult<br>o Very difficult<br>o Extremely difficult
Patient Health Questionnaire 9-item scale (PHQ-9) for Depression	Over the past 2 weeks, how often have you been bothered by any of the following problems?<br>1. Little interest or pleasure in doing things<br>2. Feeling down, depressed or hopeless<br>3. Trouble falling asleep, staying asleep, or sleeping too much<br>4. Feeling tired or having little energy<br>5. Poor appetite or overeating<br>6. Feeling bad about yourself – or that you're a failure or have let yourself or your family down

© 2022 Tordoff DM et al. *JAMA Network Open.*

	7. Trouble concentrating on things, such as reading the newspaper or watching television<br>8. Moving or speaking so slowly that other people could have noticed. Or, the opposite – being so fidgety or restless that you have been moving around a lot more than usual.<br>9. Thoughts that you would be better off dead or of hurting yourself in some way<br><u>With response options:</u> not at all, several days, over half of days, nearly everyone day, and I don't know.<br><br>10. If you checked off any problems, how difficult have those problems made it for you to do your work, take care of things at home, or get along with other people?<br>o Not difficult at all<br>o Somewhat difficult<br>o Very difficult<br>o Extremely difficult
Self-harm or Suicidal Thoughts	"Over the past 2 weeks, how often have you been bothered by thoughts that you would be better off dead or of hurting yourself in some way?" (Item-9 from the PHQ-9)
Covariates	
Mental Health Therapy	A **readiness assessment** is when the patient and their family meet with a mental health professional before starting any medical treatment. Other than having an assessment, are you receiving ongoing mental health therapy?<br>• Yes<br>• No
Tension with Caregivers	There is tension around my gender identity or gender expression… (check all that apply)<br>• …between my parents or guardians<br>• …between me and one or more of my parents or guardians<br>• …between me and my extended family<br>• Other: [open text box]<br>• None of the above
Substance Use (CRAFFT Screening Tool[1] Part A)	During the past 12 months, did you:<br>1. Drink any alcohol (more than a few sips)? (Do not count sips of alcohol taken during family or religious events)<br>2. Smoke any marijuana or hashish?<br>3. Use anything else to get high? ("Anything else" includes illegal drugs, over the counter and prescription drugs, and things that you sniff or "huff")<br><u>With response options:</u> yes, no, and I don't know
Connor-Davidson 10-item Resilience Scale (CD-RISC 10)	1. I am able to adapt when changes occur.<br>2. I can deal with whatever comes my way.<br>3. I can see the humorous side of things when I am faced with problems.<br>4. Having to cope with stress can make me stronger.<br>5. I tend to bounce back after illness, injury, or other hardships.<br>6. I believe I can achieve my goals, even if there are obstacles<br>7. Under pressure, I can focused and think clearly<br>8. I am not easily discouraged by failure<br>9. I think of myself as a strong person when dealing with life's challenges and difficulties.<br>10. I am able to handle unpleasant or painful feelings like sadness, fear and anger.<br><u>With response options:</u> not true at all, rarely true, sometimes true, often true, true nearly all the time, and I don't know.

II. Generalized Estimating Equation (GEE) Model Specification

GEE is a marginal model and models population averages (compared to mixed-effect models which are conditional and can model subject-specific effects). We specified the following GEE models to estimate the average change in the outcome variable (Y_i) at each time point (T) relative to baseline (Y_0) (Model 1) and the association between the exposure (E_i) and outcome (Model 2) adjusted for k-many baseline covariates (X_{k0}).

Model 1: $logit(Y_i) = \beta_0 + \beta_2 T_i + \beta_3 Y_0 + \sum \alpha_k X_{k0}$
Model 2: $logit(Y_i) = \beta_0 + \beta_1 E_i + \beta_2 T_i + \beta_3 Y_0 + \sum \alpha_k X_{k0}$

We allow the exposure (receipt of PB/GAH) to vary over time, where i indicates the month, and thus use an independent working correlation structure. This model assumes there are no time-varying covariates associated with the exposures and that the exposure is exogenous. A visual schematic of this model is included below in eFigure 1, the counts and percentages of participants in the exposure group at each timepoint is included in eTable 2, and the prevalence of the outcome variables over time stratified by exposure group is included in eTable 3.

eFigure 1. Schematic of Generalized Estimating Equation Model

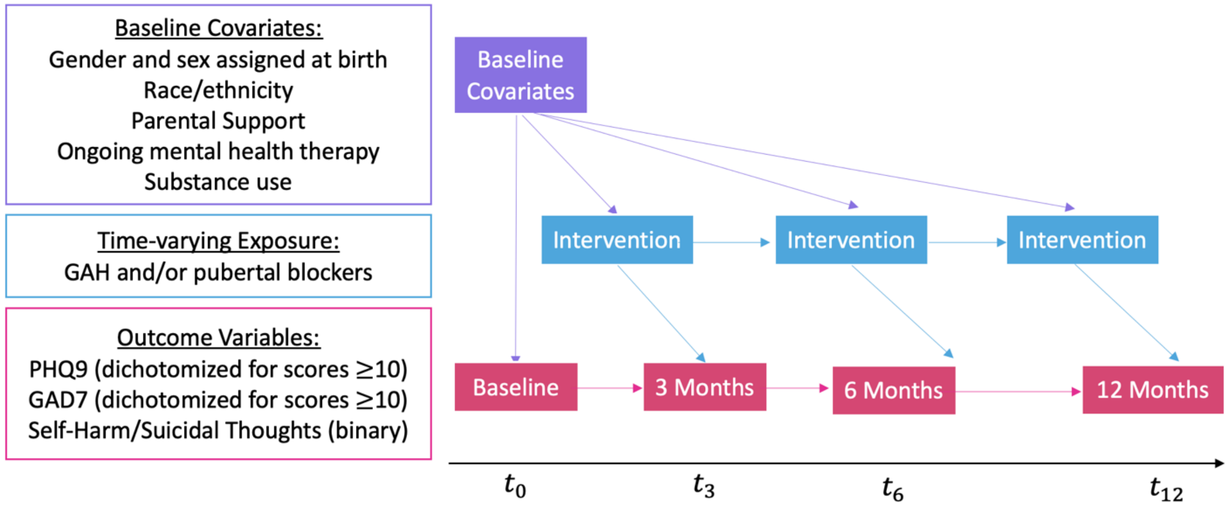

eTable 2. Prevalence of Exposure Over Time

	Baseline	3 months	6 months	12 months
N	104	84	84	65
**Exposure (no.,%)**				
PB/GAH	7 (7%)	44 (52%)	59 (71%)	57 (89%)
None	97(93%)	41 (48%)	24 (29%)	7 (11%)

eTable 3. Prevalence of Outcomes Over Time by Exposure Group

**Time Point:**	**Baseline**		**3 Months**		**6 Months**		**12 Month**	
Exposure:	*PB/GAH*	*None*	*PB/GAH*	*None*	*PB/GAH*	*None*	*PB/GAH*	*None*
N	7	92	44	38	59	24	57	6
**Outcomes (no.,%)**								
Moderate to Severe Depression	4 (57%)	54 (59%)	24 (55%)	29 (76%)	33 (56%)	13 (54%)	32 (56%)	5 (83%)
Moderate to Severe Anxiety	4 (57%)	47 (51%)	23 (52%)	23 (61%)	28 (48%)	10 (42%)	29 (51%)	4 (67%)
Self-harm or Suicidal Thoughts	3 (43%)	41 (45%)	13 (30%)	21 (55%)	25 (42%)	11 (46%)	21 (37%)	5 (83%)

© 2022 Tordoff DM et al. *JAMA Network Open.*

There were a small number of youth who did not complete the PHQ-9 or GAD-7 on each survey: 4 youth at baseline, 3 youth at 3 months, and 1 youth at 12 months.

© 2022 Tordoff DM et al. *JAMA Network Open.*

eFigure 2. Association Between Receipt of Gender-Affirming Hormones or Puberty Blockers and Mental Health Outcomes

Associations with moderate or severe depression, anxiety, and self-harm/suicidal thoughts are estimated from bivariate and multivariable GEE models

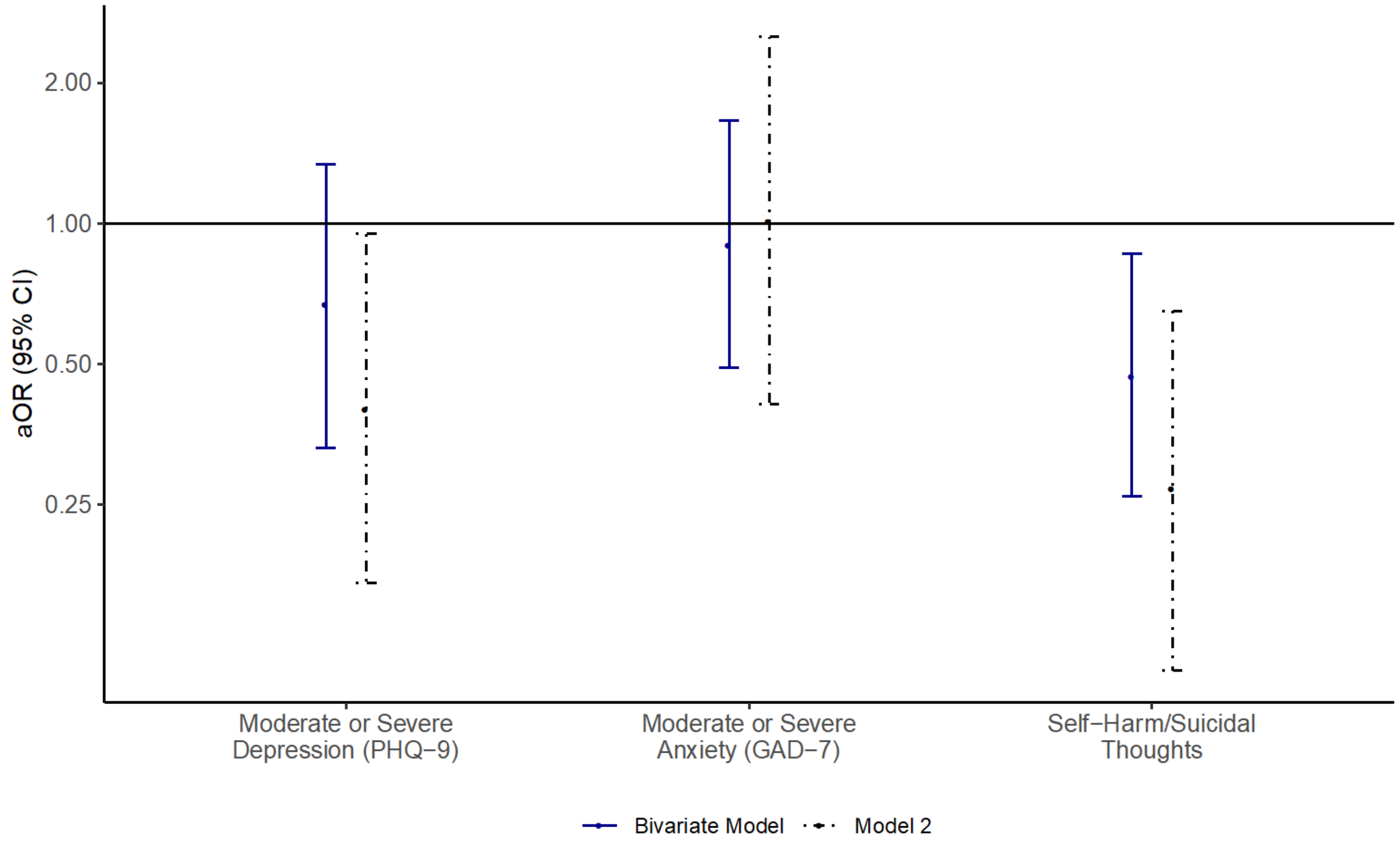

© 2022 Tordoff DM et al. *JAMA Network Open.*

III. E-Values

A. Calculation

The E-value is a relatively new measure related to the evidence for causality that can be used to assess the robustness of observational study results to unmeasured confounding.[2] It is defined as the "minimum strength of association, on the risk ratio scale, that an unmeasured confounder would need to have with both the treatment and outcome to explain away a treatment– outcome association."[3] Based on the work of VanderWeele et al.[3] the following equations can be used the estimate the E-value for an odds ratio (OR) when the outcome is common (i.e., 15% at the end of follow-up) and when the estimated OR is less than one:

$$\text{Letting } RR^* = 1/\ \text{sqrt}(OR)$$
$$\text{E-value} = RR^* + \text{sqrt}\{RR^* \times (RR^* - 1)\}$$

Applying these equations, we obtain the following E-values:

eTable 4. E-Value Calculation for Association Between Puberty Blockers or Gender-Affirming Hormones and Mental Health Outcomes

Model	Outcome	Effect Estimate (OR [95%CI])	E-value
Model 2	Moderate to Severe Depression	0.40 (0.17, 0.95)	2.56 (1.19, 4.28)
Model 2	Suicidality	0.27 (0.11, 0.65)	3.25 (1.79, 5.48)

B. Interpretation

We can interpret these findings to suggest that (1) the observed OR of 0.40 could be explained away by an unmeasured confounder that was associated with both the PB/GAH and the moderate to severe depression by a risk ratio of 2.56-fold each, above and beyond the measured confounders, but weaker confounding could not do so, and (2) the observed OR of 0.27 could be explained away by an unmeasured confounder that was associated with both the PB/GAH and the moderate to severe depression by a risk ratio of 3.25-fold each, above and beyond the measured confounders, but weaker confounding could not do so. This is evidence that our findings are robust to a moderate to high degree of unmeasured confounding, since "In the context of biomedical and social sciences research, effect sizes ≥2 or 3-fold occasionally occur but are not particularly common; a variable that affects both treatment *and* outcome each by 2- or 3-fold would likely be even less common."[3]

In observational studies, unmeasured confounding and lack of exchangeability pose the greatest barrier to drawing causal inferences from observational cohort studies. In addition, there are notable pitfalls in overly relying on p-values for the interpreting the significance of results. For instance, studies with a large sample size often have the statistical power to precisely estimate associations and obtain very small p-values; the p-value may be made arbitrarily small by increasing the sample size, even for small effect sizes. In contrast, the E-value depends on the magnitude of the association; it cannot be made arbitrarily large simply by increasing the sample size. Thus, bias adjustments, such as calculating the E-value, assess robustness of study findings to unmeasured confounding, thereby offering an important supplement to p-values.

IV. Sensitivity Analyses

A. Disaggregated Exposure Variable

We separately examined the association of PB and GAH with the outcomes of interest, although we *a priori* did not anticipate being powered to detect statistically significant associations due to our small sample size and the relatively low proportion of youth who accessed PB (n=19).

eTable 5. Examining Association Between Puberty Blockers or Gender-Affirming Hormones and Mental Health Outcomes Separately

**A. Bivariate Models**						
	**Moderate or Severe Depression (PHQ-9 ≥ 10)**		**Moderate or Severe Anxiety (GAD-7 ≥ 10)**		**Any Self-harm/Suicidal Thoughts**	
	aOR (95% CI)	P	aOR (95% CI)	P	aOR (95% CI)	P
Gender-affirming hormones	0.75 (0.36, 1.59)	0.459	0.93 (0.49, 1.78)	0.823	0.64 (0.35, 1.14)	0.131
Puberty blockers	0.52 (0.17, 1.59)	0.250	0.76 (0.29, 1.98)	0.568	0.47 (0.13, 1.69)	0.249
**B. Multivariable Models (i.e. Model 2)**						
	**Moderate or Severe Depression (PHQ-9 ≥ 10)**		**Moderate or Severe Anxiety (GAD-7 ≥ 10)**		**Any Self-harm/Suicidal Thoughts**	
	aOR (95% CI)	P	aOR (95% CI)	P	aOR (95% CI)	P
GAH	0.40 (0.16, 1.01)	0.053	1.02 (0.44, 2.37)	0.963	0.43 (0.18, 1.01)	0.052
Puberty blockers	0.52 (0.17, 1.58)	0.248	0.72 (0.26, 2.05)	0.543	0.44 (0.11, 1.74)	0.242
Time (month)						
Baseline	ref		ref		ref	
3 months	**3.34 (1.47, 7.62)**	**0.004**	1.55 (0.65, 3.67)	0.324	1.52 (0.65, 3.57)	0.333
6 months	1.89 (0.77, 4.64)	0.166	0.81 (0.31, 2.12)	0.665	2.30 (1.00, 5.27)	0.049
12 months	2.93 (0.93, 9.23)	0.067	0.99 (0.35, 2.78)	0.983	2.25 (0.64, 7.99)	0.208
Mental Health & Substance Use at Baseline						
Moderate or Severe Depression (PHQ-9 ≥ 10)	**18.2 (8.26, 39.9)**	**<0.001**	NA		NA	
Moderate or Severe Anxiety (GAD-7 ≥ 10)	**4.17 (1.97, 8.84)**	**<0.001**	**12.3 (6.16, 24.5)**	**<0.001**	NA	
Self-Harm/Suicidal Thoughts	NA		NA		**22.6 (11.6, 44.3)**	**<0.001**
Any substance use	**3.21 (1.47, 7.01)**	**0.003**	**2.19 (1.08, 4.45)**	**0.031**	1.95 (0.99, 3.83)	0.053
Resilience at Baseline (CD-RISC ≥ 22.5)[1]	NA		**0.47 (0.23, 0.94)**	**0.033**	NA	

© 2022 Tordoff DM et al. *JAMA Network Open.*

B. Restricting Analysis to Youth Age 13-17 Years Old

We restricted our analysis to minor youth age 13-17 (n=90), since they were subject to different laws related to consent and pre-requisite mental health assessments.

eTable 6. Bivariate Model Restricted to Youths Ages 13 to 17 Years

	Moderate or Severe Depression (PHQ-9 ≥ 10)[1]		Moderate or Severe Anxiety (GAD-7 ≥ 10)[2]		Any Self-harm/Suicidal Thoughts[3]	
	aOR (95% CI)	P	aOR (95% CI)	P	aOR (95% CI)	P
GAH/Puberty blockers	0.75 (0.35, 1.63)	0.473	0.79 (0.41, 1.53)	0.486	**0.47 (0.24, 0.94)**	**0.033**
Time						
Baseline	ref		ref		ref	
3 months	**2.55 (1.26, 5.17)**	**0.010**	1.19 (0.54, 2.62)	0.659	1.22 (0.56, 2.68)	0.615
6 months	1.15 (0.48, 2.75)	0.758	0.82 (0.39, 1.71)	0.596	1.29 (0.61, 2.73)	0.499
12 months	1.37 (0.48, 3.94)	0.557	0.84 (0.37, 1.90)	0.680	0.81 (0.33, 2.00)	0.649
Gender						
Transgender male or male	ref		ref		ref	
Transgender female or female	1.11 (0.48, 2.55)	0.803	1.30 (0.60, 2.82)	0.499	1.37 (0.54, 3.46)	0.511
Non-binary or genderfluid	**3.12 (1.01, 9.58)**	**0.047**	2.29 (0.55, 9.56)	0.256	**3.86 (1.11, 13.4)**	**0.033**
Race and ethnicity						
White	ref		ref		ref	
Black, Indigenous, and Persons of Color	1.19 (0.51, 2.75)	0.691	0.77 (0.38, 1.56)	0.468	0.82 (0.44, 1.54)	0.541
Age						
13-15	ref		ref		ref	
16-17	1.19 (0.51, 2.75)	0.691	0.63 (0.29, 1.39)	0.252	0.86 (0.44, 1.68)	0.657
Mental Health & Substance Use at Baseline						
Moderate or Severe Depression (PHQ-9 ≥ 10)	**31.0 (14.1, 68.3)**	**<0.001**	2.18 (0.96, 4.94)	0.063	1.23 (0.57, 2.67)	0.593
Moderate or Severe Anxiety (GAD-7 ≥ 10)	**4.97 (2.17, 11.36)**	**<0.001**	**14.0 (6.76, 29.1)**	**<0.001**	1.57 (0.80, 3.1)	0.193
Self-Harm/Suicidal Thoughts	1.26 (0.57, 2.78)	0.572	1.61 (0.76, 3.40)	0.215	**18.7 (9.72, 35.9)**	**<0.001**
Receiving mental health therapy	1.70 (0.72, 4.05)	0.228	0.72 (0.32, 1.59)	0.411	0.70 (0.30, 1.63)	0.412
Any substance use	**4.51 (1.94, 10.49)**	**<0.001**	1.83 (0.86, 3.88)	0.114	**2.47 (1.21, 5.03)**	**0.013**
Tension with Caregivers	**2.59 (1.08, 6.22)**	**0.032**	1.33 (0.62, 2.86)	0.469	1.53 (0.81, 2.89)	0.193
Resilience at Baseline (CD-RISC ≥ 22.5)[4]	0.88 (0.40, 1.89)	0.734	**0.42 (0.21, 0.87)**	**0.019**	0.70 (0.34, 1.43)	0.329

eTable 7. Multivariable Model Restricted to 90 Youths Ages 13 to 17 Years

A. Model 1 measuring temporal trends in mental health outcomes

	**Moderate or Severe Depression (PHQ-9 ≥ 10)**		**Moderate or Severe Anxiety (GAD-7 ≥ 10)**		**Any Self-harm/Suicidal Thoughts**	
	aOR (95% CI)	P	aOR (95% CI)	P	aOR (95% CI)	P
Time (month)						
Baseline	ref		ref		ref	
3 months	**2.83 (1.25, 6.44)**	**0.013**	1.22 (0.54, 2.78)	0.634	1.19 (0.53, 2.66)	0.672
6 months	1.12 (0.42, 3.00)	0.822	0.83 (0.38, 1.80)	0.638	1.27 (0.59, 2.75)	0.538
12 months	1.19 (0.37, 3.87)	0.767	0.85 (0.37, 1.93)	0.692	0.76 (0.30, 1.93)	0.558
Mental Health & Substance Use at Baseline						
Moderate or Severe Depression (PHQ-9 ≥ 10)	**24.1 (9.96, 58.2)**	**<0.001**	NA		NA	
Moderate or Severe Anxiety (GAD-7 ≥ 10)	**3.80 (1.82, 7.96)**	**<0.001**	**12.7 (6.11, 26.3)**	**<0.001**	NA	
Self-Harm/Suicidal Thoughts	NA		NA		**20.9 (10.7, 40.9)**	**<0.001**
Any substance use	**3.41 (1.41, 8.25)**	**0.006**	2.01 (0.93, 4.37)	0.077	**2.50 (1.23, 5.10)**	**0.012**
Resilience at Baseline (CD-RISC ≥ 22.5)[1]	NA		**0.40 (0.19, 0.83)**	**0.015**	NA	

B. Model 2 measuring the association between GAH/puberty blockers and mental health outcomes

	**Moderate or Severe Depression (PHQ-9 ≥ 10)**		**Moderate or Severe Anxiety (GAD-7 ≥ 10)**		**Any Self-harm/Suicidal Thoughts**	
	aOR (95% CI)	P	aOR (95% CI)	P	aOR (95% CI)	P
GAH/Puberty blockers	0.51 (0.19, 1.37)	0.182	0.84 (0.29, 2.40)	0.745	**0.32 (0.12, 0.88)**	**0.027**
Time (month)						
Baseline	ref		ref		ref	
3 months	**3.79 (1.47, 9.78)**	**0.006**	1.32 (0.49, 3.53)	0.581	1.93 (0.76, 4.88)	0.165
6 months	1.73 (0.59, 5.06)	0.315	0.93 (0.30, 2.91)	0.905	**2.58 (1.02, 6.57)**	**0.046**
12 months	2.14 (0.53, 8.73)	0.287	0.98 (0.27, 3.58)	0.979	1.99 (0.52, 7.66)	0.317
Mental Health & Substance Use at Baseline						
Moderate or Severe Depression (PHQ-9 ≥ 10)	**24.3 (9.92, 59.3)**	**<0.001**	NA		NA	
Moderate or Severe Anxiety (GAD-7 ≥ 10)	**4.01 (1.85, 8.69)**	**<0.001**	**12.7 (6.11, 26.5)**	**<0.001**	NA	
Self-Harm/Suicidal Thoughts	NA		NA		**24.3 (12.2, 48.2)**	**<0.001**
Any substance use	**3.18 (1.34, 7.55)**	**0.009**	1.98 (0.91, 4.32)	0.085	**2.33 (1.15, 4.73)**	**0.019**
Resilience at Baseline (CD-RISC ≥ 22.5)[1]	NA		**0.40 (0.19, 0.83)**	**0.015**	NA	

© 2022 Tordoff DM et al. *JAMA Network Open.*

C. Dichotomous Outcome for Depression Based on the PHQ-8

We conducted sensitivity analyses using the PHQ-8 score,[4] which is equivalent to the PHQ-9 with item-9 regarding self-harm/suicidal thoughts removed. We conducted these analyses in order to determine whether item-9 was driving any associations between moderate to severe depression since we analyzed self-harm/suicidal thoughts as a separate outcome. For these analyses we define moderate or severe depression as a PHQ-8 score ≥10.

eTable 8. Sensitivity Analyses Using Patient Health Questionnaire 8-item Scale Score of 10 or Greater for Moderate to Severe Depression

		Model 1			Model 2	
		aOR (95% CI)	P		aOR (95% CI)	P
Puberty blockers or Gender-affirming hormones		**0**	**0.039**		**0.38 (0.15, 0.98)**	**0.044**
Time (month)						
Baseline		ref			ref	
	3 months	**2.56 (1.07, 6.09)**	**0.034**		**3.95 (1.52, 10.3)**	**0.005**
	6 months	0.63 (0.27, 1.44)	0.269		1.16 (0.45, 2.99)	0.753
	12 months	0.99 (0.31, 3.16)	0.990		2.23 (0.65, 7.68)	0.205
Mental Health & Substance Use at Baseline						
	Moderate or Severe Depression (PHQ-9 ≥ 10)	**22.5 (8.93, 56.6)**	**<0.001**		**23.3 (9.07, 59.7)**	**<0.001**
	Moderate or Severe Anxiety (GAD-7 ≥ 10)	**4.31 (2.15, 8.67)**	**<0.001**		**4.57 (2.22, 9.4)**	**<0.001**
	Self-Harm/Suicidal Thoughts	NA			NA	
	Any substance use	**4.3 (1.79, 10.29)**	**0.001**		**4.08 (1.7, 9.81)**	**0.002**
Resilience at Baseline (CD-RISC ≥ 22.5)[1]		NA			NA	

© 2022 Tordoff DM et al. *JAMA Network Open.*

eReferences

1. Knight JR, Sherritt L, Shrier LA, Harris SK, Chang G. Validity of the CRAFFT Substance Abuse Screening Test Among Adolescent Clinic Patients. *Arch Pediatr Adolesc Med*. 2002;156(6):607-614. doi:10.1001/ARCHPEDI.156.6.607
2. Haneuse S, Vanderweele TJ, Arterburn D. Using the E-Value to Assess the Potential Effect of Unmeasured Confounding in Observational Studies. *JAMA*. 2019;321(6):602-603. doi:10.1001/JAMA.2018.21554
3. VanderWeele T, Ding P. Sensitivity Analysis in Observational Research: Introducing the E-Value. *Ann Intern Med*. 2017;167(4):268-274. doi:10.7326/M16-2607
4. Kroenke K, Strine TW, Spitzer RL, Williams JBW, Berry JT, Mokdad AH. The PHQ-8 as a measure of current depression in the general population. *J Affect Disord*. 2009;114(1-3):163-173. doi:10.1016/J.JAD.2008.06.026

© 2022 Tordoff DM et al. *JAMA Network Open.*
